# Usefulness of tissue inhibitor of metalloproteinase 1 as a predictor of sustained remission in patients with antineutrophil cytoplasmic antibody-associated vasculitis

**DOI:** 10.1186/s13075-021-02471-5

**Published:** 2021-03-20

**Authors:** Jun Ishizaki, Ayako Takemori, Kenta Horie, Daisuke Hiraoka, Koichiro Suemori, Takuya Matsumoto, Ken-ei Sada, Koichi Amano, Masayoshi Harigai, Yoshihiro Arimura, Hirofumi Makino, Katsuto Takenaka, Nobuaki Takemori, Hitoshi Hasegawa, for the Research Committee of Intractable Vasculitis Syndrome and the Research Committee of Intractable Renal Disease of the Ministry of Health, Labour and Welfare of Japan, Yohko Murakawa, Eri Muso, Atsushi Komatsuda, Satoshi Ito, Takao Fujii, Atsushi Kawakami, Izaya Nakaya, Takao Saito, Takafumi Ito, Nobuhito Hirawa, Masahiro Yamamura, Masaaki Nakano, Kosaku Nitta, Makoto Ogura, Taio Naniwa, Shoichi Ozaki, Junichi Hirahashi, Noriyoshi Ogawa, Tatsuo Hosoya, Takashi Wada, Satoshi Horikoshi, Yasushi Kawaguchi, Taichi Hayashi, Masaharu Yoshida, Tsuyoshi Watanabe, Daijo Inaguma, Kazuhiko Tsuruya, Noriyuki Homma, Tsutomu Takeuchi, Naoki Nakagawa, Shinichi Takeda, Ritsuko Katabuchi, Masayuki Iwano, Tatsuya Atsumi, Shoichi Fujimoto, Shogo Banno, Takahiko Sugihara, Masaki Kobayashi, Kunihiro Yamagata, Sakae Homma, Hiroaki Dobashi, Naotake Tsuboi, Akihiro Ishizu, Hitoshi Sugiyama

**Affiliations:** 1 Department of Hematology, Clinical Immunology, and Infectious Diseases, Ehime University Graduate School of Medicine, Toon, Ehime 791-0295 Japan; 2 Division of Analytical Bio-Medicine, Advanced Research Support Center, Ehime University, Toon, Ehime Japan; 3 Department of Clinical Epidemiology, Kochi Medical School, Kochi, Japan; 4 Department of Rheumatology and Clinical Immunology, Saitama Medical Center, Saitama Medical University, Saitama, Japan; 5 Department of Rheumatology, Tokyo Women’s Medical University School of Medicine, Tokyo, Japan; 6 Department of Nephrology and Rheumatology, Kyorin University School of Medicine, Tokyo, Japan; 7 Department of Internal Medicine, Kichijoji Asahi Hospital, Tokyo, Japan; 8 Okayama University, Okayama, Japan

**Keywords:** Antineutrophil cytoplasmic antibody-associated vasculitis, Tissue inhibitor of metalloproteinase 1, Maintenance therapy, Remission, Biomarker

## Abstract

**Background:**

We previously identified tissue inhibitor of metalloproteinase 1 (TIMP-1) as a biomarker of disease activity that distinguished mildly or highly active antineutrophil cytoplasmic antibody (ANCA)-associated vasculitis (AAV) from remission 6 months after the initiation of remission-induction therapy. In the present study, we investigated whether TIMP-1 is clinically useful as a predictor of relapse and sustained remission in AAV patients with microscopic polyangiitis (MPA) and granulomatosis with polyangiitis (GPA) during maintenance therapy.

**Methods:**

The relationship between serum TIMP-1 levels and clinical outcomes in AAV patients receiving maintenance therapy was assessed using the follow-up data of a Japanese large-cohort study (the RemIT-JAV-RPGN study) and data collected from AAV patients on maintenance therapy in our hospital (the MAAV-EU study).

**Results:**

In the RemIT-JAV RPGN study, serum levels of TIMP-1 were significantly higher in mildly active AAV patients with MPA and GPA 6 months after the initiation of remission-induction therapy than in patients in remission. Regarding maintenance therapy, elevated levels of TIMP-1 in patients in remission were associated with relapse and/or difficulty reducing the glucocorticoid dosage after 6 to 12 months. In the MAAV-EU study, serum levels of TIMP-1 were elevated in relapsed patients 6 months before relapse, earlier than the increase in serum levels of CRP. Analyses of both studies revealed that approximately 30% of patients in remission with a serum TIMP-1 level ≥ 150 ng/mL relapsed after 6 to 12 months, while the majority of patients with a TIMP-1 level < 150 ng/mL sustained remission for at least 12 months.

**Conclusion:**

We herein demonstrated that TIMP-1 is more useful as a predictive biomarker of sustained remission than as a predictor of relapse in maintenance therapy for AAV. TIMP-1 levels < 150 ng/mL are important for the long-term maintenance of remission and may be an indicator for the tapering or cessation of treatment.

**Supplementary Information:**

The online version contains supplementary material available at 10.1186/s13075-021-02471-5.

## Background

Antineutrophil cytoplasmic antibody (ANCA)-associated vasculitis (AAV) comprises three distinct diseases: granulomatosis with polyangiitis (GPA), microscopic polyangiitis (MPA), and eosinophilic granulomatosis with polyangiitis (EGPA) [1]. Clinical relapse following remission is common in AAV [2]. ANCA and traditional acute-phase indicators, including C-reactive protein (CRP), are used in clinical practice as biomarkers of disease activity and predictors of relapse. However, these biomarkers lack the sensitivity and specificity for monitoring the disease activity of AAV [3–7]. Several circulating biomarkers for monitoring disease activity or predicting relapse have been reported in large-cohort studies and systematic literature searches [8–15]; however, none have been applied to clinical practice. Furthermore, the identification of predictors of sustained remission is important for treatment planning.

We previously identified promising biomarkers of disease activity and organ involvement in AAV using a targeted proteomics approach with serum samples collected in a Japanese nationwide large cohort study (RemIT-JAV-RPGN) [16, 17]. In a quantitative analysis of 135 biomarker candidates, tissue inhibitor of metalloproteinase 1 (TIMP-1) was the best-performing marker of disease activity that distinguished mildly or highly active AAV from remission 6 months after the initiation of remission-induction therapy. In contrast, other markers, such as CRP and myeloperoxidase (MPO)-ANCA, did not significantly differ between patients with mild activity and in remission. Monach et al. examined 28 markers associated with inflammation, angiogenesis, tissue damage, and repair in patients enrolled in the Rituximab in AAV (RAVE) study, and suggested that TIMP-1 distinguished active AAV from remission more accurately than ESR and CRP [10]. The findings of these two large-cohort studies suggest the potential of TIMP-1 for monitoring disease activity. However, TIMP-1 was only evaluated at two points, i.e., before and 6 months after the initiation of remission-induction therapy. Therefore, detailed analysis of TIMP-1 levels during the clinical course of AAV patients is needed to verify whether TIMP-1 is clinically useful as a predictor of relapse and sustained remission during maintenance therapy.

In the present study, we excluded EGPA from subjects because of its particularities, such as eosinophil inflammation and differences in the definition of relapse [18, 19], and analyzed patients with MPA and GPA. We examine the relationship between serum levels of TIMP-1 at 6 months in patients in remission and clinical outcomes up to 18 months in the Japanese RemIT-JAV-RPGN cohort study. We also performed a serial analysis of serum TIMP-1 levels in AAV patients receiving maintenance therapy at our hospital, and investigated whether TIMP-1 is useful as a predictor of relapse and sustained remission.

## Methods

### Healthy donors and patients

In an analysis using follow-up data from the RemIT-JAV-RPGN study, we assessed 69 AAV patients 6 months after treatment initiation [16]. The RemIT-JAV-RPGN study is a multi-center cohort. Among 69 patients, 54 were enrolled in the RemIT-JAV-RPGN study and 15 were patients in our hospital. All patients were newly diagnosed with AAV and fulfilled the criteria for primary systemic vasculitis proposed by the European Medicine Agency (EMEA) algorithm [20], according to which the 69 patients were diagnosed as follows: 46 with MPA and 23 with GPA.

In serial analysis of serum TIMP-1 levels, we assessed 30 AAV patients receiving maintenance therapy in our hospital who were followed up for more than 12 months (the MAAV-EU study; Maintenance therapy for AAV in the Ehime University study). These patients were diagnosed as follows: 16 with MPA and 14 with GPA. All patients had achieved remission when they were enrolled in the present study. The observation period was between December 2017 and March 2020.

Patients in the RemIT-JAV-RPGN and MAAV-EU studies received remission-induction therapy and maintenance therapy based on the discretion of the site investigators according to the Japanese Ministry of Health, Labour, and Welfare (MHLW) Guidelines for the Treatment of AAV. In both studies, patients younger than 20 years and those with EGPA and/or malignancy were excluded. EGPA was excluded because of its unique characteristics, such as eosinophil inflammation, and differences in organ damage, ANCA positivity, the definition of relapse and treatment responses from MPA and GPA [18, 19]. Malignancy was excluded because of its influence on the selection of treatment and serum levels of TIMP-1.

Serum samples were also obtained from 52 healthy donors.

### Outcome measures

Details of the RemIT-JAV-RPGN study protocol were reported previously [16]. Follow-up data, including vital status, Birmingham Vasculitis Activity Score (BVAS), laboratory data, and treatment, were collected at 3, 6, 12, and 18 months of treatment and at the time of relapse. Disease activity was evaluated according to BVAS version 3 [21]. In the analysis using the follow-up data of the RemIT-JAV-RPGN study, remission 6 months after the initiation of treatment was defined as BVAS 0 on two occasions at least 1 month apart according to the EULAR recommendations [22]. Sustained remission was defined as the maintenance of remission from 6 to 18 months after the initiation of treatment. Patients with difficulties reducing the glucocorticoid (GC) dosage were defined as a daily GC dosage of ≥ 10 mg prednisolone 18 months after treatment.

In the MAAV-EU study, sustained remission was defined as BVAS 0 from the time of enrollment to the end of the observation period (more than 12 months in remission). Baseline data for patients in sustained remission were obtained 6 months after enrollment, whereas those for relapsed patients were collected at the time of relapse. We compared serum levels of TIMP-1 and CRP between both groups 6 months (− 6 M), 3 months (− 3 M), and 1 month (− 1 M) before baseline and at baseline.

In both studies, relapse was defined as the re-occurrence or new onset of clinical signs and symptoms attributable to active vasculitis.

### Enzyme-linked immunosorbent assays (ELISA)

Serum in patients in the RemIT-JAV-RPGN study was analyzed before and 6 months after the initiation of treatment. In the MAAV-EU study, each biomarker was analyzed using serum samples serially collected every 1 to 3 months during the observation period. Serum samples were frozen at − 80 °C until used.

Samples were analyzed using commercially available ELISA kits according to the manufacturer’s instructions. The following ELISA kits were used: CRP, TIMP-1, matrix metalloproteinase 3 (MMP-3), and C-X-C motif chemokine ligand 13 (CXCL13) (R&D Systems, Minneapolis, MN, USA); MPO-ANCA and proteinase-3 (PR3)-ANCA (MBL, Nagoya, Japan).

### Statistical analysis

We used baseline and follow-up data at 6, 12, and 18 months and at relapse in the RemIT-JAV-RPGN study. Values were expressed as medians and interquartile ranges (IQR) or as numbers and percentages. Continuous non-parametric variables were compared using the Mann-Whitney *U* test between 2 groups and the Kruskal-Wallis test and Steel-Dwass test among 3 groups and categorical variables using Fisher’s direct probability test. The distinction of active AAV from remission in the same patients with AAV was compared using the Wilcoxon signed-rank test. The receiver operating characteristic (ROC) curves were constructed using logistic regression to assess the ability of the biomarker and define the optimal cutoff point. The area under the ROC curve (AUC) was calculated. The optimal cutoff point was determined using the Youden index, which is defined as the maximum sum of sensitivity and specificity. Correlations between paired data were analyzed using Spearman’s rank correlation.

A multivariable analysis was performed using a logistic regression model to adjust for confounding factors, including age, sex, AAV type, and BVAS before treatment. Relapse-free survival was analyzed using the Kaplan-Meier method and compared using the Log-rank test. Differences at *p* <  0.05 were considered to be significant. Statistical analyses were performed using JMP software, version 15 (SAS Institute, Cary, NC, USA).

## Results

### Patient characteristics and relationship between serum TIMP-1 levels and clinical outcomes at 6 months in the RemIT-JAV-RPGN study

The characteristics of the 69 patients enrolled in the RemIT-JAV-RPGN study and in our hospital at baseline and 6 months after the initiation of treatment are shown in Table 1. Among these patients, 52 (75%) were in remission at 6 months, whereas 17 (25%) were not and had mildly active AAV (median BVAS 5 [IQR3-6]). The serum level of creatinine and estimated glomerular filtration rate (eGFR) in patients before treatment and at 6 months did not significantly differ between the two groups (before treatment, creatinine: *p* = 0.063, eGFR: *p* = 0.063; at 6 months, creatinine: *p* = 0.14, eGFR: *p* = 0.055). The serum levels of TIMP-1 in patients before treatment did not correlate with the serum level of creatinine or eGFR (vs creatinine: *ρ* = 0.01, *p* = 0.92; vs eGFR: *ρ* = − 0.04, *p* = 0.76).

**Table 1:** Clinical characteristics of 69 patients at baseline and 6 months after the initiation of treatment in the RemIT-JAV-RPGN study

	All patients (*n* = 69)	Remission (*n* = 52)	Not in remission (*n* = 17)
Male/female, n/n	27/42	22/30	5/12
Age, years	69 (61–77)	69 (61–77)	67 (61–73)
GPA/MPA, n/n	23/46	16/36	7/10
MPO-ANCA-positive	59 (86%)	45 (87%)	14 (82%)
PR3-ANCA-positive	12 (17%)	8 (15%)	4 (24%)
ANCA-negative	2 (3%)	2 (4%)	0 (0%)
Serum creatinine at baseline, mg/dL	0.92 (0.66–1.86)	0.88 (0.66–1.37)	1.86 (0.69–4.53)
Serum creatinine at 6 months, mg/dL	0.91 (0.74–1.28)	0.9 (0.74–1.08)	1.26 (0.81–1.47)
eGFR at baseline, mL/min/1.73 m^2^	54 (22–69)	60 (34–70)	22 (11–67)
eGFR at 6 months, mL/min/1.73 m^2^	53 (39–67)	61 (42–71)	41 (29–53)
Disease activity and organ involvement			
BVAS score	15 (12–22)	15 (12–22)	19 (12–22)
BVAS general-positive	52 (75%)	41 (79%)	11 (65%)
BVAS cutaneous-positive	11 (16%)	8 (15%)	3 (18%)
BVAS mucous membranes/eyes-positive	11 (16%)	10 (19%)	1 (6%)
BVAS ENT-positive	28 (41%)	21 (40%)	7 (41%)
BVAS chest-positive	28 (41%)	20 (38%)	8 (47%)
BVAS cardiovascular-positive	4 (6%)	4 (8%)	0 (0%)
BVAS abdominal-positive	1 (1%)	1 (2%)	0 (0%)
BVAS renal-positive	53 (77%)	40 (77%)	13 (76%)
BVAS nervous system-positive	23 (33%)	16 (31%)	7 (41%)
Induction therapy, 0–6 months			
Glucocorticoids, mg/day	45 (30–50)	43 (30–50)	45 (40–50)
Glucocorticoid pulse	28 (41%)	18 (35%)	10 (59%)
Cyclophosphamide	38 (55%)	27 (52%)	11 (65%)
MPO-ANCA negative conversion at 6 months^a^	39 (70%)	29 (69%)	10 (71%)
PR3-ANCA negative conversion at 6 months^b^	7 (58%)	4 (50%)	3 (75%)

Values are medians (IQR) or *n* (%)

Four patients had double (MPO and PR3) positive ANCA

^a^The rate of MPO-ANCA negative conversion was only calculated for MPO-ANCA-positive patients whose MPO-ANCA levels were measured at 6 months (all patients, *n* = 56; remission, *n* = 42; not in remission, *n* = 14)

^b^The rate of PR3-ANCA negative conversion was only calculated for PR3-ANCA-positive patients whose PR3-ANCA levels were measured at 6 months (all patients, *n* = 12; remission, *n* = 8; not in remission, *n* = 4)

GPA, granulomatosis with polyangiitis; MPA, microscopic polyangiitis; MPO, myeloperoxidase; PR3, proteinase-3; ANCA, antineutrophil cytoplasmic antibody; eGFR, estimated glomerular filtration rate; BVAS, Birmingham Vasculitis Activity Score version 3; ENT, ear, nose, and throat

**Fig. 1:**
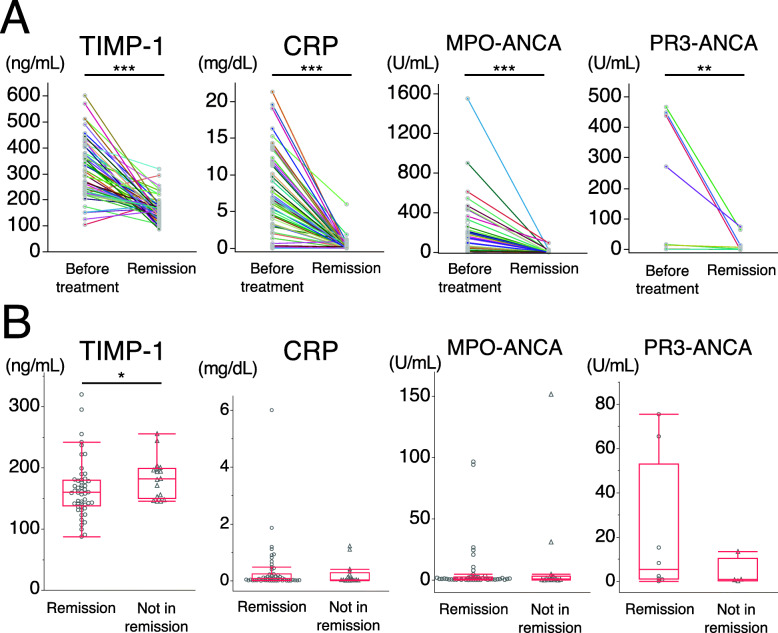
Marker levels in AAV patients before and 6 months after the initiation of treatment. **a** Comparison of marker levels between highly active AAV before treatment and remission 6 months after treatment. The titers of markers were measured in paired serum samples (before and 6 months after the initiation of treatment) from 52 patients with AAV [36 microscopic polyangiitis (MPA) and 16 granulomatosis with polyangiitis (GPA)]. Each line connects data obtained on one patient. MPO-ANCA refers to only MPO-ANCA-positive patients (*n* = 45). PR3-ANCA refers to only PR3-ANCA-positive patients (*n* = 8). **b** Comparison of marker levels between patients in remission (*n* = 52) and those not in remission (*n* = 17) 6 months after the initiation of treatment. The 17 patients not in remission (10 MPA and 7 GPA) had mildly active AAV (median BVAS 5, range 3–6). MPO-ANCA only refers to MPO-ANCA-positive patients (remission, *n* = 45; not in remission, *n* = 14). PR3-ANCA only refers to PR3-ANCA-positive patients (remission, *n* = 8; not in remission, *n* = 4). Each dot represents one patient. Box plots show the median and IQR. Whiskers indicate the most extreme points within 1.5-fold of the IQR of the box. *p < 0.05. **p < 0.01. ***p < 0.001. AAV, antineutrophil cytoplasmic antibody-associated vasculitis; TIMP-1, tissue inhibitor of metalloproteinase 1; CRP, C-reactive protein; MPO-ANCA, myeloperoxidase-antineutrophil cytoplasmic antibody; PR3-ANCA, proteinase-3-antineutrophil cytoplasmic antibody

As shown in Fig. 1a, serum levels of TIMP-1, CRP, and MPO-ANCA were significantly higher in patients before treatment than in those in remission at 6 months. Serum levels of TIMP-1 were significantly higher in patients before treatment than in healthy controls (302 [227–381] ng/mL vs 133 [122–150] ng/mL, *p* <  0.001). In addition, PR3-ANCA was significantly higher in patients before treatment than in those in remission at 6 months; however, the number (*n* = 8) of PR3-ANCA-positive patients was small. Serum levels of TIMP-1 were significantly higher in the 17 patients not in remission than in the 52 in remission at 6 months (182 [152–197] ng/mL vs 160 [139–179] ng/mL) (Fig. 1b). After adjustments for potential confounders, such as age, sex, and AAV type, using multivariable analysis, an elevated level of TIMP-1 (per 20 ng/mL increase) was still significantly associated with no remission (odds ratio [OR] 1.38 [95% CI 1.03–1.84], *p* = 0.024). To distinguish between these 2 groups, the TIMP-1 cutoff level for remission at 6 months was 144 ng/mL with a sensitivity of 38% and specificity of 100%. In contrast, CRP, MPO-ANCA, and PR3-ANCA did not significantly differ between the 2 groups. These results suggest that TIMP-1 is a superior biomarker to CRP, MPO-ANCA, and PR3-ANCA for monitoring the disease activities of MPA and GPA.

### Relationship between serum TIMP-1 levels and clinical outcomes from 6 to 18 months in the RemIT-JAV-RPGN study

**Fig. 2:**
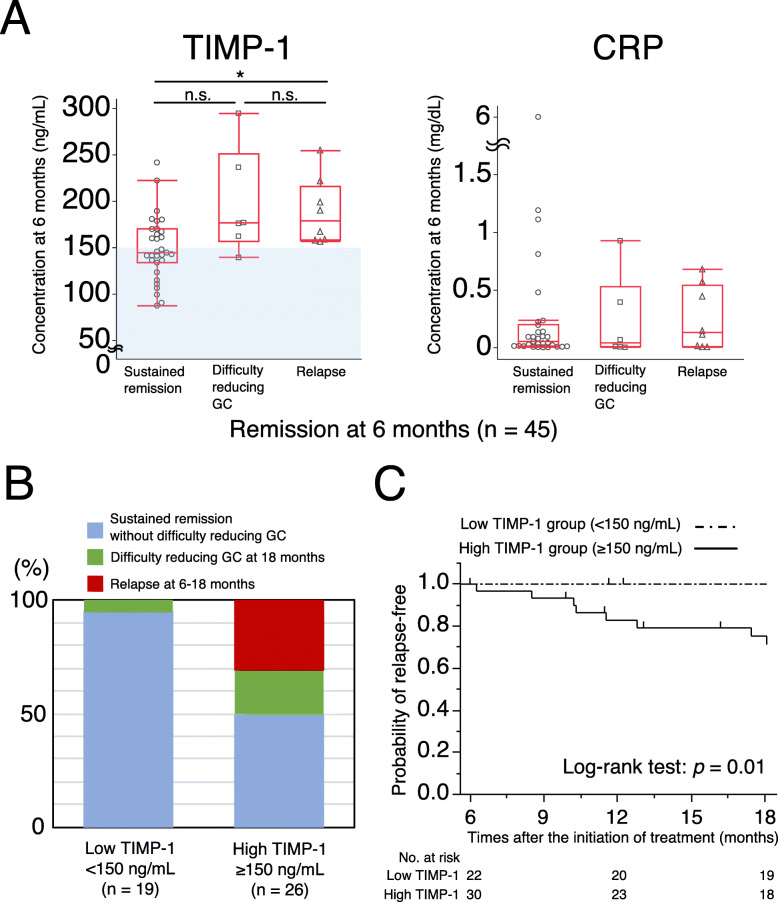
Relationship between serum TIMP-1 levels and clinical outcomes from 6 to 18 months in the RemIT-JAV-RPGN study. **a** Relationship between serum TIMP-1 and CRP levels at 6 months and clinical outcomes from 6 to 18 months in 45 patients in remission 6 months after treatment (31 microscopic polyangiitis and 14 granulomatosis with polyangiitis). Difficulty reducing GC refers to patient receiving a daily GC dosage of ≥ 10 mg prednisolone 18 months after treatment. Each dot represents one patient. The light blue background indicates a low TIMP-1 level (< 150 ng/mL). Box plots show the median and IQR. Whiskers indicate the most extreme points within 1.5-fold of the IQR of the box. *p < 0.05. n.s. Not significant. **b** Comparison of clinical outcomes from 6 to 18 months in the low TIMP-1 group (< 150 ng/mL, *n* = 19) and high TIMP-1 group (≥ 150 ng/mL, *n* = 26). **c** Kaplan-Meier curves for the probability of remaining relapse-free in the low TIMP-1 group (< 150 ng/mL, *n* = 22) and high TIMP-1 group (≥ 150 ng/mL, *n* = 30). The tick mark indicates each censored case. Significance was measured using the Log-rank test. TIMP-1, tissue inhibitor of metalloproteinase 1; CRP, C-reactive protein; GC, glucocorticoid

We assessed the clinical outcomes of maintenance therapy from 6 to 18 months among the 52 patients in remission; 45 (31 with MPA and 14 with GPA) were followed up for at least 18 months. Of the 7 patients lost to the follow-up by 18 months, 2 died from causes other than vasculitis and 5 had missing follow-up data or interrupted visits during the observation period. Eight (16%) patients, 6 with MPA and 2 with GPA, relapsed by 18 months: 5 from 6 to 12 months and 3 from 12 to 18 months (Table 2, Fig. 2a). The median GC dosage at 18 months in all patients in remission was 7.5 (IQR 5–9) mg prednisolone, and 11 patients, including 5 relapsed patients, had difficulty reducing the GC dosage (median 13 [IQR 10–15.5] mg). The use of immunosuppressants from 6 to 18 months did not significantly differ between patients with difficulty reducing GC and those in sustained remission without difficulty reducing GC. A significant difference was observed in TIMP-1 levels at 6 months among the following 3 groups: sustained remission, difficulty reducing GC, and relapse, by the Kruskal-Wallis test (*p* = 0.014), but not in CRP. TIMP-1 levels were significantly higher in the relapse group than in the sustained remission group.

**Table 2:** Clinical outcomes in patients receiving maintenance therapy in the RemIT-JAV-RPGN study

	Relapse and/or difficulty reducing GC (*n* = 14)	Sustained remission without difficulty reducing GC (*n* = 31)	*p* value^d^
Male/female, n/n	9/5	9/22	0.047
Age, years	68 (61–72)	68 (61–77)	0.60
GPA/MPA, n/n	3/11	11/20	0.49
MPO-ANCA-positive before treatment	13 (93%)	25 (81%)	0.41
PR3-ANCA-positive before treatment	1 (7%)	7 (23%)	0.4
BVAS score before treatment	18 (11–25)	15 (12–18)	0.31
Glucocorticoids, mg/day			
At 6 months	15 (11–18)	13 (10–17)	0.19
At 12 months	12 (10–14)	7 (5–10)	< 0.001
At 18 months	11 (10–15)	5 (5–8)	< 0.001
Immunosuppressants	7 (50%)	18 (58%)	0.75
AZA/MTX/others, *n*	4/1/2	13/0/10	
Serum creatinine at 6 months, mg/dL	1.00 (0.83–2.09)	0.86 (0.70–1.02)	0.053
eGFR at 6 months, mL/min/1.73 m^2^	43 (21–74)	61 (46–72)	0.3
CRP at 6 months, mg/dL	0.09 (0.006–0.43)	0.05 (0.01–0.17)	0.96
MPO-ANCA at 6 months, U/mL^a^	1.3 (1–2)	1 (0.4–2.6)	0.42
MPO-ANCA negative conversion at 6 months^b^	10 (83%)	14 (61%)	0.48
TIMP-1 at 6 months, ng/mL	176 (159–216)	144 (135–168)	0.004
High TIMP-1 at 6 months^c^	13 (93%)	13 (42%)	0.003

Values are medians (IQR) or *n* (%)

^a^Only MPO-ANCA-positive patients whose MPO-ANCA levels were measured at 6 months (relapse and/or difficulty reducing GC, *n* = 13; sustained remission without difficulty reducing GC, *n* = 25)

^b^The rate of MPO-ANCA negative conversion was only calculated for MPO-ANCA-positive patients whose MPO-ANCA levels were measured at 6 months (relapse and/or difficulty reducing GC, *n* = 12; sustained remission without difficulty reducing GC, *n* = 23)

^c^Serum TIMP-1 levels at 6 months ≥ 150 ng/mL

^d^*p* < 0.05 was considered to be significant

GC, glucocorticoids; GPA, granulomatosis with polyangiitis; MPA, microscopic polyangiitis; MPO, myeloperoxidase; PR3, proteinase-3; ANCA, antineutrophil cytoplasmic antibody; BVAS, Birmingham Vasculitis Activity Score version 3; AZA, azathioprine; MTX, methotrexate; eGFR, estimated glomerular filtration rate; TIMP-1, tissue inhibitor of metalloproteinase 1

Age, the AAV type, BVAS before treatment, the serum level of creatinine, eGFR, and the use of immunosuppressants from 6 to 18 months were not significantly different between patients with relapse and/or difficulty reducing GC and those in sustained remission without difficulty reducing GC. The proportion of males was significantly higher among patients with relapse and/or difficulty reducing GC than among those in sustained remission without difficulty reducing GC. Serum levels of TIMP-1 at 6 months were significantly higher in patients with relapse and/or difficulty reducing GC than in those in sustained remission without difficulty reducing GC (*p* = 0.004). The cutoff point for sustained remission without difficulty reducing GC was 148 ng/mL with a sensitivity of 58%, specificity of 93%, and AUC of 0.77. In contrast, serum levels of CRP and MPO-ANCA at 6 months did not significantly differ between both groups. Based on healthy control data and this cutoff point, we divided patients into high (≥ 150 ng/mL at 6 months after treatment) and low (< 150 ng/mL) TIMP-1 groups. In a multivariable analysis of variables including sex and the presence of elevated TIMP-1 levels at 6 months (TIMP-1 ≥ 150 ng/mL or < 150 ng/mL), elevated TIMP-1 levels at 6 months were significantly associated with patients with relapse and/or with difficulty reducing GC from 6 to 18 months (OR 14.1 [95% CI 1.6–125.5], *p* = 0.003) (Table 3).

**Table 3:** Logistic regression analysis of patients with relapse and/or difficulty reducing glucocorticoids from 6 to 18 months

Variable	Unit	Adjusted OR (95% CI)	*p* value^a^
Sex	Male	2.7 (0.6–12.1)	0.18
High TIMP-1 at 6 months	≥ 150 ng/mL	14.1 (1.6–125.5)	0.003

^a^*p* < 0.05 was considered to be significant

OR, odds ratio; CI, confidence intervals; TIMP-1, tissue inhibitor of metalloproteinase 1

We compared clinical outcomes from 6 to 18 months between the high and low TIMP-1 groups (Fig. 2b). Among 45 patients, 26 and 19 were divided into the high and low TIMP-1 groups, respectively. There were 8 (31%) patients with relapse, 5 (19%) with difficulty reducing GC, and 13 (50%) in remission without difficulty reducing GC in the high TIMP-1 group. On the other hand, all patients in the low TIMP-1 group (95%), except for one with difficulty reducing GC, sustained remission. Relapse-free survival was significantly higher in the low TIMP-1 group than in the high TIMP-1 group (Fig. 2c). These findings suggest that patients with TIMP-1 levels < 150 ng/mL on maintenance therapy had more strongly suppressed disease activity and may be in remission for 12 months.

### Serial analysis of serum TIMP-1 levels and clinical outcomes in the MAAV-EU study

**Fig. 3:**
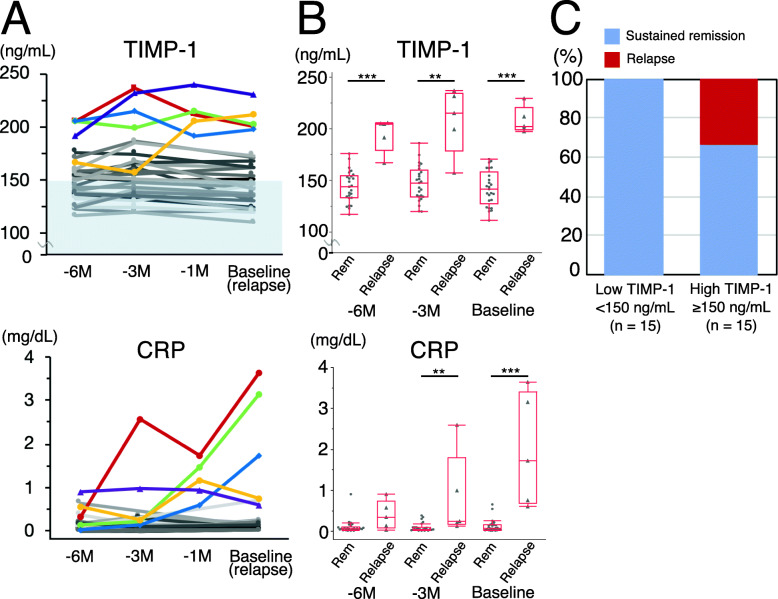
Relationship between serum TIMP-1 levels and clinical outcomes in the MAAV-EU study. **a** Serial analysis of serum TIMP-1 and CRP levels in 30 patients [16 microscopic polyangiitis (MPA) and 14 granulomatosis with polyangiitis (GPA)] with AAV receiving maintenance therapy. All patients achieved remission at the time of enrollment. Color lines present relapsed patients (*n* = 5) and gray lines patients in sustained remission (*n* = 25). Baseline refers to 6 months after enrollment in patients in sustained remission and at the time of relapse in relapsed patients. The light blue background indicates a low TIMP-1 level (< 150 ng/mL). **b** Comparison of serum TIMP-1 and CRP levels between sustained remission patients (*n* = 25) and relapsed patients (*n* = 5) in both groups 6 and 3 months before baseline and at baseline. Each dot represents one patient. Box plots show the median and IQR. Whiskers indicate the most extreme points within 1.5-fold of the IQR of the box. **c** Comparison of clinical outcomes in the low TIMP-1 group (< 150 ng/mL 6 months before baseline, *n* = 15) and high TIMP-1 group (≥ 150 ng/mL, *n* = 15). **p < 0.01. ***p < 0.001. TIMP-1, tissue inhibitor of metalloproteinase 1; CRP, C-reactive protein; − 6 M, − 6 months; − 3 M, − 3 months; − 1 M, − 1 month; Rem, sustained remission

To confirm the results obtained, a more detailed serial analysis of TIMP-1 levels and clinical outcomes was performed in AAV patients receiving maintenance therapy in our hospital using serum samples and clinical data collected every 1 to 3 months (MAAV-EU study). The characteristics of 30 AAV patients in the MAAV-EU study were shown in Table 4. The median disease duration of all patients was 2.3 (IQR 1.5–8.5) years. Five out of the 30 patients (17%), 2 with MPA and 3 with GPA, relapsed, while 25 (83%) sustained remission during the observation period (more than 12 months) (Fig. 3a). Of the 5 relapsed patients, 3 had first relapse and the other 2 had second relapse. Characteristics, such as age, sex, and disease duration, the serum level of creatinine, and eGFR did not significantly differ between relapsed patients and patients with sustained remission. Furthermore, no significant differences were observed in the GC dosage at 6 months before baseline and the combination rate of immunosuppressants, whereas GC dosages at baseline and 3 months before baseline were significantly higher in relapsed patients. As shown in Fig. 3b, serum levels of TIMP-1 and CRP at baseline and 3 months before baseline were significantly higher in relapsed patients, whereas only TIMP-1 levels 6 months before baseline were significantly higher in relapsed patients. The median serum TIMP-1 level in relapsed patients 6 months before relapse was 205 (191–205) ng/mL. This result indicates that TIMP-1 levels predict the possibility of relapse earlier than CRP during maintenance therapy. The serum level of TIMP-1 at the baseline did not correlate with the serum level of creatinine or eGFR (vs creatinine: *ρ* = 0.2, *p* = 0.29; vs eGFR: *ρ* = − 0.12, *p* = 0.51).

**Table 4:** Comparison of patients with relapse and sustained remission in the MAAV-EU study

	Relapse (*n* = 5)	Sustained remission (*n* = 25)	*p* value^b^
Male/female, n/n	2/3	10/15	1.0
Age, years	68 (65–71)	66 (61–72)	0.40
Disease duration, year	1.9 (1.1–2.1)	3.5 (1.5–11.7)	0.062
Previous history of relapse	2 (40%)	7 (28%)	0.62
GPA/MPA, n/n	3/2	11/14	0.64
MPO-ANCA-positive at onset	4 (80%)	16 (64%)	0.64
PR3-ANCA-positive at onset	1 (20%)	7 (28%)	1.0
Serum creatinine at baseline, mg/dL	0.75 (0.59–0.92)	1.0 (0.69–0.95)	0.34
eGFR at baseline, mL/min/1.73 m^2^	78 (63–80)	65 (50–68)	0.22
MPO-ANCA negative conversion at enrollment^a^	3 (75%)	8 (53%)	0.60
Glucocorticoids at − 6 months, mg/day	10 (9–10)	5 (5–10)	0.36
Glucocorticoids at − 3 months, mg/day	9 (9–10)	5 (5–8)	0.035
Glucocorticoids at baseline, mg/day	9 (8–10)	5 (5–6)	0.019
Immunosuppressants	3 (60%)	18 (72%)	0.62

Values are medians (IQR) or *n* (%)

^a^The rate of MPO-ANCA negative conversion was only calculated for MPO-ANCA-positive patients whose MPO-ANCA levels were measured at enrollment (relapse, *n* = 4; sustained remission, *n* = 15)

^b^*p* < 0.05 was considered to be significant

MAAV-EU study, maintenance therapy for antineutrophil cytoplasmic antibody-associated vasculitis in the Ehime University study; GPA, granulomatosis with polyangiitis; MPA, microscopic polyangiitis; MPO, myeloperoxidase; PR3, proteinase-3; ANCA, antineutrophil cytoplasmic antibody; eGFR, estimated glomerular filtration rate

We compared clinical outcomes between the high (≥ 150 ng/mL 6 months before baseline) and low (< 150 ng/mL) TIMP-1 groups in the MAAV-EU study. Thirty patients were equally divided into the high and low TIMP-1 groups. As shown in Fig. 3c, 5 (33%) patients in the high TIMP-1 group relapsed after 6 months. In contrast, all patients in the low TIMP-1 group sustained remission during the observation period.

Among the 20 MPO-ANCA-positive patients at onset in the MAAV-EU study, 15 showed the negative conversion of MPO-ANCA before enrollment, whereas 5 did not. Of the 15 patients with the negative conversion of MPO-ANCA, 4 and 1 showed the reappearance of MPO-ANCA at enrollment and 1 month after enrollment, respectively, while the remaining 10 remained MPO-ANCA negative. One (25%) out of the 4 patients with relapse and 4 (36%) out of the 11 without relapse showed the reappearance of MPO-ANCA. The reappearance rate of MPO-ANCA did not significantly differ between the two groups. All 5 patients without negative conversion did not relapse. Among the 8 PR3-ANCA-positive patients at onset, 5 showed the negative conversion of PR3-ANCA at enrollment and 3 did not. All 5 patients with the negative conversion of PR3-ANCA remained negative for PR3-ANCA and maintained remission. One out of the 3 patients without the negative conversion of PR3-ANCA relapsed.

Analyses of the RemIT-JAV-RPGN and MAAV-EU studies revealed that approximately 30% of patients in remission with a serum TIMP-1 level ≥ 150 ng/mL relapsed after 6 to 12 months, while the majority of patients with a TIMP-1 level < 150 ng/mL sustained remission for at least 12 months. Therefore, a serum TIMP-1 level < 150 ng/mL reflects complete remission, while that of ≥ 150 ng/mL indicates subclinical inflammation, even with BVAS 0, during maintenance therapy in AAV.

### Serum levels of MMP-3 and CXCL13 in the RemIT-JAV-RPGN and MAAV-EU studies

In addition to TIMP-1, MMP-3 and CXCL13 have been identified as useful biomarkers that discriminate active AAV from remission [10]. We examined the serum levels of MMP-3 and CXCL13 in both studies.

**Fig. 4:**
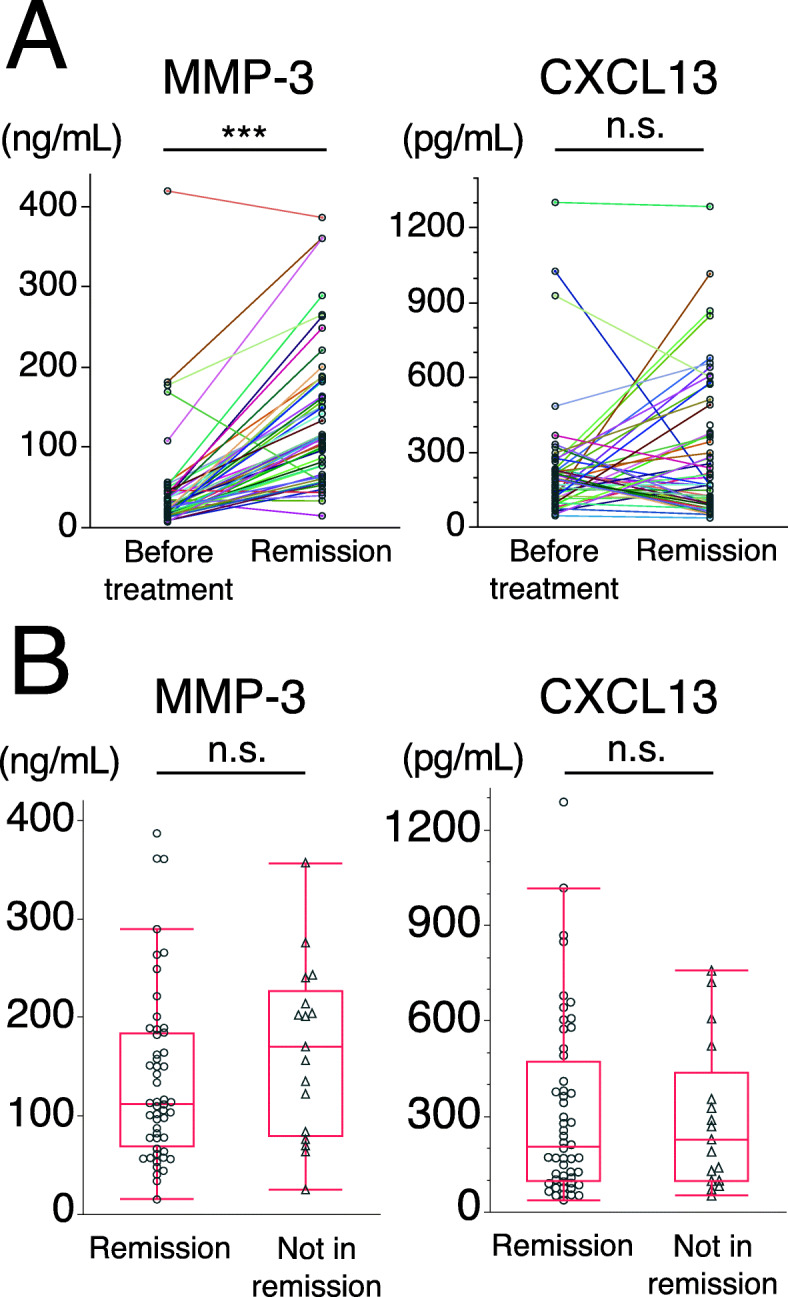
Serum levels of MMP-3 and CXCL13 in AAV patients before and 6 months after the initiation of treatment. **a** Comparison of MMP-3 and CXCL13 levels between highly active AAV before treatment and remission 6 months after treatment. The titers of markers were measured in paired serum samples (before and 6 months after the initiation of treatment) from 52 patients with AAV [36 microscopic polyangiitis (MPA) and 16 granulomatosis with polyangiitis (GPA)]. Each line connects data obtained on one patient. **b** Comparison of MMP-3 and CXCL13 levels between patients in remission (*n* = 52) and those not in remission (*n* = 17) 6 months after the initiation of treatment. The 17 patients not in remission (10 MPA and 7 GPA) had mildly active AAV (median BVAS 5, range 3–6). Each dot represents one patient. Box plots show the median and IQR. Whiskers indicate the most extreme points within 1.5-fold of the IQR of the box. ***p < 0.001. n.s. Not significant. AAV, antineutrophil cytoplasmic antibody-associated vasculitis

In the RemIT-JAV-RPGN study, the serum levels of MMP-3 and CXCL13 were significantly higher in patients before treatment than in healthy controls (MMP-3: 29.6 [16.7–46.3] ng/mL vs 16.5 [11.0–24.6] ng/mL, *p* <  0.001; CXCL13: 189.4 [113.6–242.4] pg/mL vs 67.6 [48.8–94.6] pg/mL, *p* <  0.001). As shown in Fig. 4a, the serum levels of MMP-3 were significantly higher in patients in remission at 6 months than in those before treatment. Serum levels of CXCL13 did not significantly differ between the groups of patients before treatment and in remission at 6 months and increased in 27 (52%) out of 52 patients in remission at 6 months. MMP-3 and CXCL13 levels did not significantly differ between patients in remission and those not in remission at 6 months (Fig. 4b). Serum levels of MMP-3 in 52 patients in remission at 6 months correlated with the GC dosage at 6 months (*ρ* = 0.51, *p* < 0.001), whereas those of CXCL13 did not. A significant difference was observed in MMP-3 levels at 6 months among the following 3 groups: sustained remission, difficulty reducing GC, and relapse, by the Kruskal-Wallis test (*p* = 0.003), but not in CXCL13. MMP-3 levels were significantly higher in the difficulty reducing GC group than in the other 2 groups (See Supplementary Figure 1, Additional file 1).

In the MAAV-EU study, serum levels of MMP-3 and CXCL13 at baseline and 3 and 6 months before baseline did not significantly differ between relapsed patients and patients with sustained remission (See Supplementary Figure 2, Additional file 1).

## Discussion

Relapse is common in AAV and requires repeated remission-induction therapy [2]. Although the definition of clinical remission may appear to be straightforward, many patients in remission still exhibit persistent inflammatory and immunological activities. BVAS is a common clinical indicator for evaluating disease activity in AAV, and remission is defined as a BVAS score of 0 [21]. However, true remission may not be achieved in many patients with BVAS 0 and persistent inflammation may increase the risk of subsequent relapse.

ANCA is often used to evaluate disease activity; however, its utility for predicting relapse is limited [2, 7, 23–27]. Large cohorts identified PR3-ANCA, but not MPO-ANCA, as an independent risk factor for relapse [23–26]. In the serial analysis of PR3-ANCA in the RAVE study, an elevated PR3-ANCA titer was a poor predictor of subsequent relapse within the overall population, but was predictive of relapse in patients with renal disease or alveolar hemorrhage [26]. In two prospective Japanese cohorts, the reappearance of MPO-ANCA was significantly associated with relapse, and was particularly useful for predicting relapse in patients with renal involvement [27]. However, among 25 relapsed patients after negative conversion of MPO-ANCA, 6 (24%) patients did not experience reappearance of MPO-ANCA. On the other hand, among 76 patients without negative conversion of MPO-ANCA, 60 patients (79%) maintained remission. Based on these findings, ANCA shows limited utility for predicting relapse and is rarely used as a predictor of sustained remission. In the MAAV-EU study, the rate of reappearance after the negative conversion of MPO-ANCA did not significantly differ between the groups of patients in relapse and not in relapse. Watanabe et al. examined new-onset patients who were in remission 6 months after induction therapy and reported that 84% of patients with relapse had renal involvement [27]. On the other hand, in the MAAV-EU study that analyzed patients receiving maintenance therapy with a median disease duration of 2.3 years, only 1 out of 5 patients with relapse had renal involvement. These discrepancies in the results obtained may be attributed to differences in the subject groups.

Previous studies investigated predictive markers of relapse other than ANCA. In the RAVE study, an increase in serum calprotectin levels by 2 or 6 months was predictive of relapse by 18 months in PR3-ANCA-positive AAV patients treated with rituximab. However, this marker was not useful in patients treated with oral cyclophosphamide [14]. The CD8 T-cell transcription signature was associated with subsequent relapse in AAV or systemic lupus erythematosus, but requires further prospective validation [28].

In the two large cohort studies, the Japanese RemIT-JAV-RPGN study (our previous study) [17] and RAVE study [10], TIMP-1 levels were a useful biomarker for distinguishing mildly or highly active AAV from remission 6 months after the initiation of remission-induction therapy regardless of differences in the population, such as AAV type, ANCA type, and treatment. In our previous study, TIMP-1 correlated with the total BVAS score before treatment and was less likely to be elevated in bacterial infections than CRP. In contrast, CRP, ESR, and MPO-ANCA were unable to distinguish patients with mildly active AAV from those in remission.

In the present study, we examined whether TIMP-1 levels are clinically useful as a predictor of relapse and sustained remission during maintenance therapy. Based on the results of an 18-month follow-up in the RemIT-JAV-RPGN cohort study, elevated TIMP-1 levels at 6 months were associated with relapse and/or difficulty reducing GC from 6 to 18 months. In the MAAV-EU study, TIMP-1 levels were significantly higher in relapsed patients 6 months before relapse than in sustained remission patients. Based on these findings, many patients with relapse have elevated TIMP-1 levels 6 months before relapse. However, elevated TIMP-1 levels were observed in some patients in sustained remission.

Since the cutoff point for sustained remission without difficulty reducing GC was 148 ng/mL, patients in remission were divided into two groups of TIMP-1 levels, ≥ 150 ng/mL and < 150 ng/mL, and their relationship with clinical outcomes was examined. In the analysis of both studies, approximately 30% of patients in remission with a TIMP-1 level ≥ 150 ng/mL relapsed after 6 to 12 months. More importantly, the majority of patients with a TIMP-1 level < 150 ng/mL, which meant a decrease to a level approximating that in healthy controls, remained in remission for at least 12 months. Therefore, a serum TIMP-1 level < 150 ng/mL reflects complete remission, whereas ≥ 150 ng/mL indicates subclinical inflammation. Moreover, TIMP-1 levels are more useful as a predictor of sustained remission than of relapse. During maintenance therapy, TIMP-1 levels < 150 ng/mL are an important indicator of the long-term maintenance of remission. Thus, in patients with AAV, the maintenance of a TIMP-1 level < 150 ng/mL may allow for the tapering or cessation of treatment.

TIMP-1 is expressed by various cell types in most human tissues. It is an endogenous inhibitor of MMPs and its production increases in response to an increase in MMPs. In addition, it is a well-known regulator of extracellular matrix turnover, tissue remodeling, inflammation, cell growth, apoptosis, and cellular behavior in tissue [29–31]. Therefore, TIMP-1 levels may more accurately reflect the condition of inflammatory sites than CRP; however, the underlying mechanisms have not yet been elucidated. The normalization of TIMP-1 levels may indicate that vascular inflammation has completely subsided. On the other hand, serum levels of TIMP1 were previously reported to be elevated in patients with various cancers, myocardial infarction, ischemic stroke, and sepsis [29–36]. Therefore, it is important to note that TIMP-1 may not serve as a predictor of sustained remission in AAV patients with these disorders.

Monach et al. suggested that TIMP-1, MMP-3, and CXCL13 distinguished active AAV from remission more accurately than ESR and CRP in patients enrolled in the RAVE study [10]. Similar to the analysis in the RAVE study, serum levels of MMP-3 and CXCL13 in the present study were significantly higher in new-onset active AAV patients before treatment than in healthy controls. However, MMP-3 and CXCL13 were not useful for distinguishing between active AAV and remission 6 months after the initiation of treatment or for evaluating disease activity in patients receiving maintenance therapy. This is because glucocorticoids increase the serum levels of MMP-3 and CXCL13 regardless of disease activity in AAV patients. Although the underlying mechanisms remain unclear, previous studies reported that glucocorticoids increased serum MMP-3 levels [37–39] and also that glucocorticoids were associated with elevated CXCL13 levels in multiple diseases, including vasculitis [40]. In AAV patients in the RAVE study, 85% of those in remission discontinued prednisolone. Therefore, serum MMP-3 and CXCL13 levels were affected less by glucocorticoids and reflected disease activity. In contrast, neither marker accurately reflected disease activity in the present study because all patients, except for one at 6 months, in the RemIT-JAV-RPGN study and all patients in the MAAV-EU study received glucocorticoids. The present study also suggested that both disease activity and glucocorticoids increased MMP-3 and CXCL13 levels, which may limit their clinical usefulness as activity markers.

The present study has several limitations. The sample size was small in both studies. We validated the results obtained across two different cohorts of AAV, which is a rare disease; however, validation in a larger cohort is needed in the future. Furthermore, in the analysis of clinical outcomes in the Remit-JAV-RPGN study, 7 patients were lost to the follow-up by 18 months due to death, were missing follow-up data, or had interrupted visits during the observation period. Moreover, since the number of PR3-ANCA-positive patients was small in both studies, comparative analysis with PR3-ANCA could not be performed sufficiently. The small number is because MPA is the predominant type among patients with AAV in Japan and more than 80% of Japanese AAV patients are positive for pANCA/MPO-ANCA [16, 41, 42]. Since these limitations are often difficult to avoid in clinical research involving rare diseases, such as AAV, we expect TIMP-1 to become a predictor of sustained remission during maintenance therapy and contribute to treatment strategies for AAV.

## Conclusions

We assessed the usefulness of serum TIMP-1 levels as a predictive biomarker of relapse and sustained remission in AAV patients during maintenance therapy. Analyses of the RemIT-JAV-RPGN and MAAV-EU studies revealed that serum TIMP-1 levels in relapsed patients were elevated 6 months before relapse, earlier than the increase in serum levels of CRP. More importantly, the majority of patients with a TIMP-1 level < 150 ng/mL maintained remission for at least 12 months, indicating complete remission. Therefore, TIMP-1 levels are more useful as a predictive biomarker of sustained remission than as a predictor of relapse in maintenance therapy for AAV. TIMP-1 levels < 150 ng/mL are an important indicator of the long-term maintenance of remission and treatment strategies.

## Supplementary Information

**Additional file 1: Supplementary Figure 1.** Relationship between serum MMP-3 and CXCL13 levels and clinical outcomes from 6 to 18 months in the RemIT-JAV-RPGN study. **Supplementary Figure 2.** Relationship between serum MMP-3 and CXCL13 levels and clinical outcomes in the MAAV-EU study.

## Data Availability

All data generated or analyzed during this study are included in this published article.

## Abbreviations

AAV: Antineutrophil cytoplasmic antibody-associated vasculitis
ANCA: Antineutrophil cytoplasmic antibody
BVAS: Birmingham vasculitis activity score version 3
CRP: C-reactive protein
CXCL13: C-X-C motif chemokine ligand 13
EGPA: Eosinophilic granulomatosis with polyangiitis
EMEA: European Medicine Agency
GC: Glucocorticoid
GPA: Granulomatosis with polyangiitis
IQR: Interquartile range
MAAV-EU study: Maintenance therapy for AAV in the Ehime University study
MHLW: The Japanese Ministry of Health, Labour, and Welfare
MMP: Matrix metalloproteinase
MPA: Microscopic polyangiitis
MPO: Myeloperoxidase
PR3: Proteinase-3
RAVE study: The Rituximab in ANCA-Associated Vasculitis study
RemIT-JAV-RPGN: Cohort study of remission induction therapy in Japanese patients with AAV and rapidly progressive glomerulonephritis
ROC: Receiver operating characteristic
TIMP-1: Tissue inhibitor of metalloproteinase-1
