# Supplementary material for: Usefulness of tissue inhibitor of metalloproteinase 1 as a predictor of sustained remission in patients with antineutrophil cytoplasmic antibody-associated vasculitis
Source: Arthritis Res Ther. 2021 Mar 20;23:91. doi: 10.1186/s13075-021-02471-5 (PMC7980538; doi:10.1186/s13075-021-02471-5)
Supplement: Supplementary file 1 — Additional file 1: Supplementary Figure 1. Relationship between serum MMP-3 and CXCL13 levels and clinical outcomes from 6 to 18 months in the RemIT-JAV-RPGN study. Supplementary Figure 2. Relationship between serum MMP-3 and CXCL13 levels and clinical outcomes in the MAAV-EU study. [file 13075_2021_2471_MOESM1_ESM.pdf]

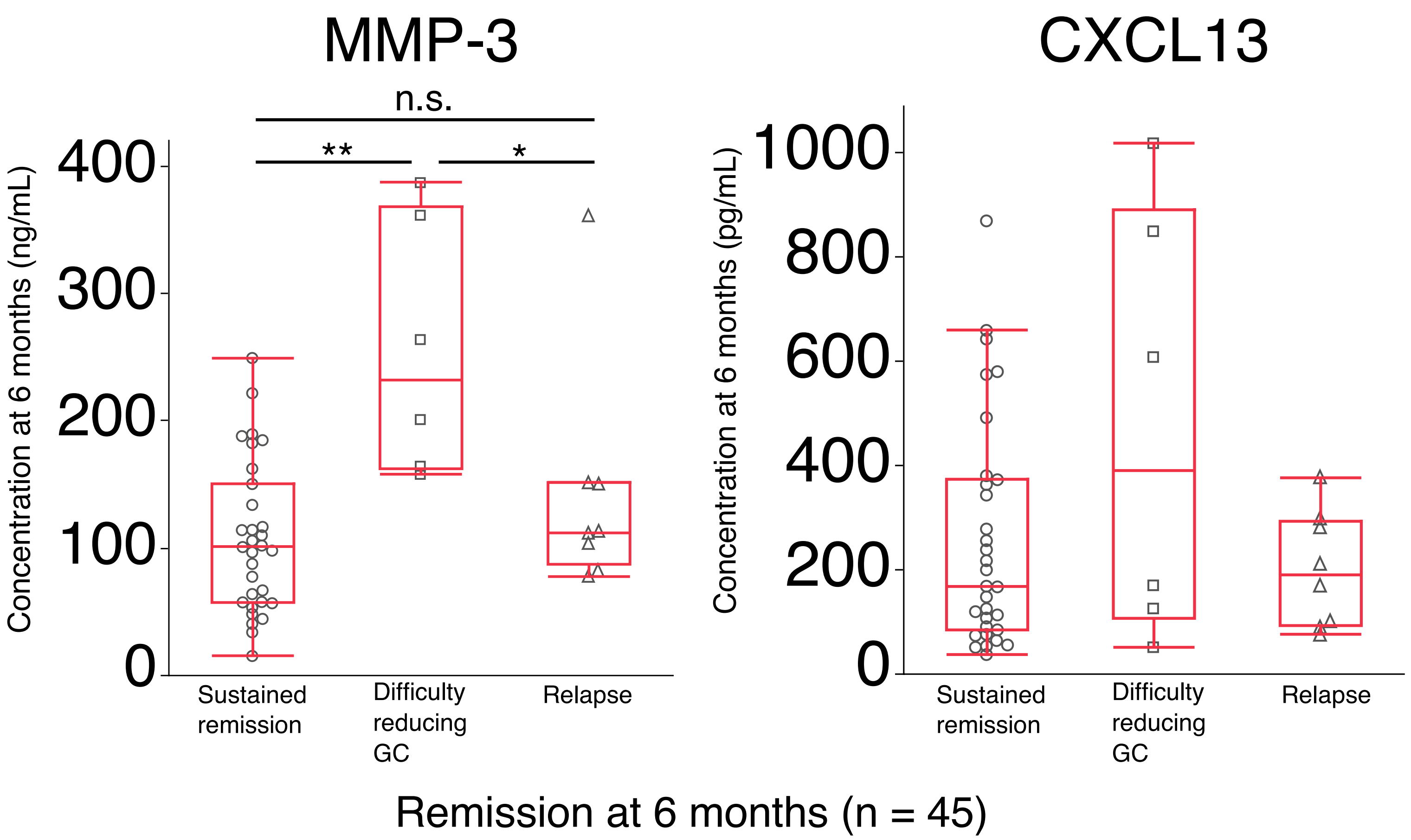

Supplementary Figure 1. Relationship between serum MMP-3 and CXCL13 levels and clinical outcomes from 6 to 18 months in the RemIT-JAV-RPGN study.

Relationship between serum MMP-3 and CXCL13 levels at 6 months and clinical outcomes from 6 to 18 months in 45 patients in remission 6 months after treatment (31 microscopic polyangiitis and 14 granulomatosis with polyangiitis). Difficulty reducing GC refers to patient receiving a daily GC dosage of ≥10 mg prednisolone 18 months after treatment. Box plots show the median and IQR. Whiskers indicate the most extreme points within 1.5-fold of the IQR of the box. *$p < 0.05$. **$p < 0.01$. n.s. Not significant.

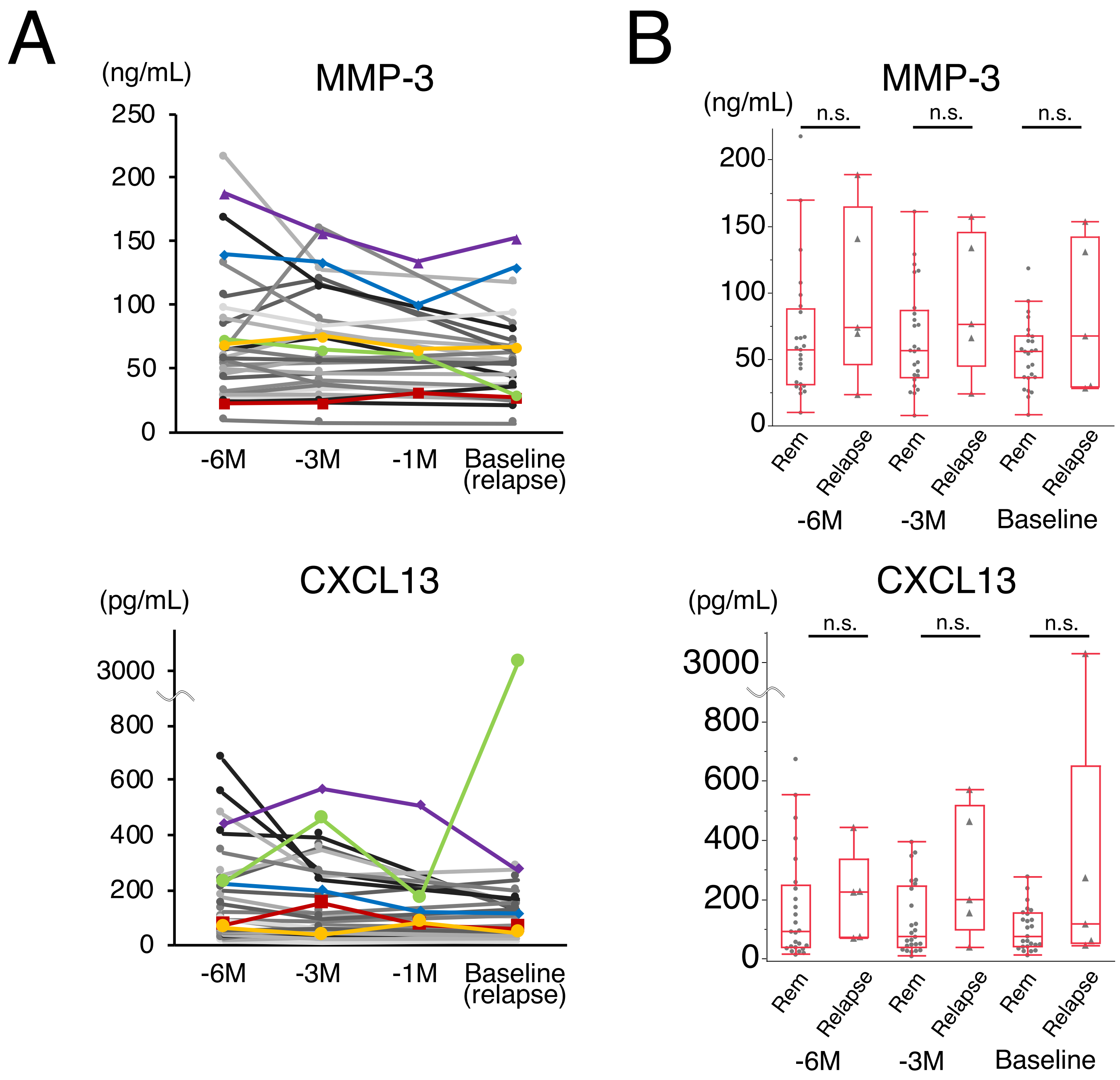

Supplementary Figure 2. Relationship between serum MMP-3 and CXCL13 levels and clinical outcomes in the MAAV-EU study.

(A) Serial analysis of serum MMP-3 and CXCL13 levels in 30 patients [16 microscopic polyangiitis (MPA) and 14 granulomatosis with polyangiitis (GPA)] with AAV receiving maintenance therapy. All patients achieved remission at the time of enrollment. Color lines present relapsed patients (n = 5) and grey lines patients in sustained remission (n =25). Baseline refers to 6 months after enrollment in patients in sustained remission and at the time of relapse in relapsed patients. (B) Comparison of serum MMP-3 and CXCL13 levels between sustained remission patients (n = 25) and relapsed patients (n = 5) in both groups 6 and 3 months before baseline and at baseline. Each dot represents one patient. Box plots show the median and IQR. Whiskers indicate the most extreme points within 1.5-fold of the IQR of the box. n.s. Not significant. -6M, -6 months; -3M, -3 months; -1M, -1 month; Rem, Sustained remission
